# Emotion-focused coping mediates the relationship between COVID-related distress and compulsive buying

**DOI:** 10.1371/journal.pone.0274458

**Published:** 2022-09-15

**Authors:** Lilla Nóra Kovács, Eva Katzinger, Sunghwan Yi, Zsolt Demetrovics, Aniko Maraz, Gyöngyi Kökönyei

**Affiliations:** 1 Institute of Psychology, ELTE Eötvös Loránd University, Budapest, Hungary; 2 Institut für Psychologie, Humboldt-Universität zu Berlin, Berlin, Germany; 3 University of Guelph, Guelph, Canada; 4 Centre of Excellence in Responsible Gaming, University of Gibraltar, Gibraltar, Gibraltar; 5 SE-NAP2 Genetic Brain Imaging Migraine Research Group, Hungarian Brain Research Program, Semmelweis University, Budapest, Hungary; Semmelweis University: Semmelweis Egyetem, HUNGARY

## Abstract

**Background and aims:**

COVID-19 posits psychological challenges worldwide and has given rise to nonadaptive behavior, especially in the presence of maladaptive coping. In the current study, we assessed whether the relationship between COVID-related distress and compulsive buying is mediated by task-focused and emotion-focused coping. We also examined whether these associations were invariant over time as the pandemic unfolded.

**Methods:**

Self-report surveys were administered online in the United States in the first six months of the pandemic (March–October 2020) in sampling batches of 25 participants every three days, resulting in a total sample of N = 1,418 (40% female, mean age = 36.6). We carried out structural equation modeling to assess whether the relationship between distress related to COVID-19 and compulsive buying is mediated by task-focused and emotion-focused coping. Time was used as a grouping variable based on events related to the pandemic in the U.S. to calculate model invariance across three time periods.

**Results:**

The results indicated significant mediation between distress, emotion-focused coping, and compulsive buying, but not between task-focused coping and compulsive buying. The mediation model showed excellent fit to the data (χ² = 1119.377, df = 420, RMSEA = 0.059 [0.055–0.064], SRMR = 0.049, CFI = 0.951, TLI = 0.947). Models were not invariant across the three examined time periods.

**Conclusions:**

Our results indicate that compulsive buying is more likely to occur in relation to emotion-focused coping as a response to COVID-related distress than in relation to task-focused coping, especially during periods of increasing distress. However, model paths varied during the course of the pandemic.

## Introduction

### COVID-19 as a chronic worldwide stressor

Coronavirus disease caused by the SARS-CoV-2 virus (the COVID-19 pandemic) has affected many aspects of daily human life, leading to increased distress and serious mental health issues in society [1–3]. The threat of epidemics to physical health, as well as the related restrictive measures, can act as triggers of distress [4], thus the COVID-19 pandemic can be considered a chronic worldwide stressor [5]. Stress is widely studied due to its considerable impact on people’s wellbeing [6]. In the current study, we focused primarily on the psychological aspect of stress—that is, the subjective feeling of distress and the coping response. This approach is in line with Lazarus and Folkman’s definition of stress—that is, the mismatch between perceived external challenges and the individual’s perceived ability to overcome them [7].

### Coping strategies in response to stress

Empirical findings suggest that the psychological reaction demonstrated in response to a stressor plays a bigger part in adaptation than the magnitude of the external stressor itself [7, 8]. Different coping strategies can be employed in response to stressors. Coping comprises the individual’s mental and behavioral attempts to manage, diminish, or endure stress [9]. Three main types of coping have been identified: task-focused, emotion-focused, and avoidance coping [10]. Task-focused coping takes the form of a proactive response to a stressful event; it seeks an optimal resolution to the problem and is associated with a sense of control [11]. Avoidance coping refers to an individual’s engagement in cognitive, behavioral, and often maladaptive activities in an attempt to divert their attention away from the stressor and deny, minimize, or otherwise avoid dealing directly with the stressful situation [12]. Within this framework, behaviors such as going out with friends, alcohol and substance use, gambling, video gaming, or even compulsive buying can be considered as means of avoidance coping. On the other hand, these behaviors may also be motivated by many other factors (e.g., temporary promotions [13]) or may serve other functions than avoidance, such as escapism [14] or seeking social support [15].

Emotion-focused coping entails responses that are focused on the self, such as emotional responses to a stressful situation, self-preoccupation, and fantasizing/daydreaming [16]. However, the wide scope of emotion-focused coping has often generated ambiguous results in terms of whether the emotion-focused coping is adaptive or not [17]. Although emotion-focused coping can be considered adaptive (e.g., positive reappraisal, the seeking of social support) when it actively facilitates emotional processing and expression, which may help to mitigate the individual’s negative reactions to the stressor, it can be considered maladaptive (e.g., denial, self-blame, or counterfactual thinking) when it encourages passivity and avoidance and therefore does not facilitate acceptance or problem solving [17]. In the current study, we adopted the latter conceptualization of emotion-focused coping, as it appears to be closely related to maladaptive behavioral outcomes such as compulsive buying [18].

### Coping strategies during COVID-19

Like many other aspects of life, the coping strategies employed during the COVID-19 pandemic have changed substantially. For instance, although seeking social support has been identified as an adaptive way to cope with distress [19, 20], this coping strategy was not readily available during social distancing. The increased rate of reported loneliness indicates that video calls may not be able to fully substitute in-person social interactions, especially in the long term [21]. Similarly, a recent study found that lockdown measures, fewer interpersonal interactions, and voluntary self-quarantine were associated with a higher incidence of mental health problems, underscoring the detrimental effects of weakened social support [22]. Moreover, it is likely that the lack of in-person social support, coupled with uncertainty about the future and fear of the disease, have all contributed to the use of coping strategies that are less adaptive as the pandemic has unfolded [4]. In line with this, the empirical results indicate that avoidance and emotion-focused coping were highly associated with symptoms of depression and anxiety during COVID-19, whereas task-focused coping was not [23].

### Compulsive buying during COVID-19

Compulsive buying involves a preoccupation with buying or urges to buy that are experienced as intrusive and uncontrollable [24]. Compulsive buying is associated with negative affectivity and is likely to result in social, personal, and/or financial difficulties [25]. According to a meta-analysis, the prevalence of compulsive buying behavior is 4.9% in adult representative samples, and even higher among younger adults and women [26]. The cognitive-behavioral model of compulsive buying [25] suggests that internal and external triggers, such as negative emotions, depression, and anxiety, may increase compulsive buying. These factors, together with distress, have become pertinent during the COVID-19 pandemic [2, 4, 5, 22, 27] and the empirical results confirm that vulnerability to compulsive buying has increased during COVID-19 due to distress. More specifically, experiencing COVID-19 symptoms and fearing COVID-19 were weakly but significantly associated with compulsive buying [28, 29]. In line with this, empirical evidence indicates that compulsive buying, together with other maladaptive behaviors such as alcohol and substance use, gambling, smoking, and binge eating, also steadily increased during the first six months of the pandemic [30]. However, further investigation is needed to ascertain the role of mediating factors between distress and compulsive buying during COVID-19.

In summary, most of the population worldwide have been experiencing elevated distress in their daily lives due to COVID-19 and the related restrictive measures [4, 5]. Chronic stress is known to contribute to the increased use of maladaptive behaviors, including compulsive buying, that may help to divert the individual’s attention away from the stressor, at least in the short term [31]. People who tend to use maladaptive coping strategies may be at increased risk of developing maladaptive behaviors [32–34], thus investigating the relationship between COVID-related distress, coping strategies, and maladaptive behavioral outcomes such as compulsive buying is of the utmost importance during the COVID-19 pandemic.

### Aims of the current study

**Fig 1:**
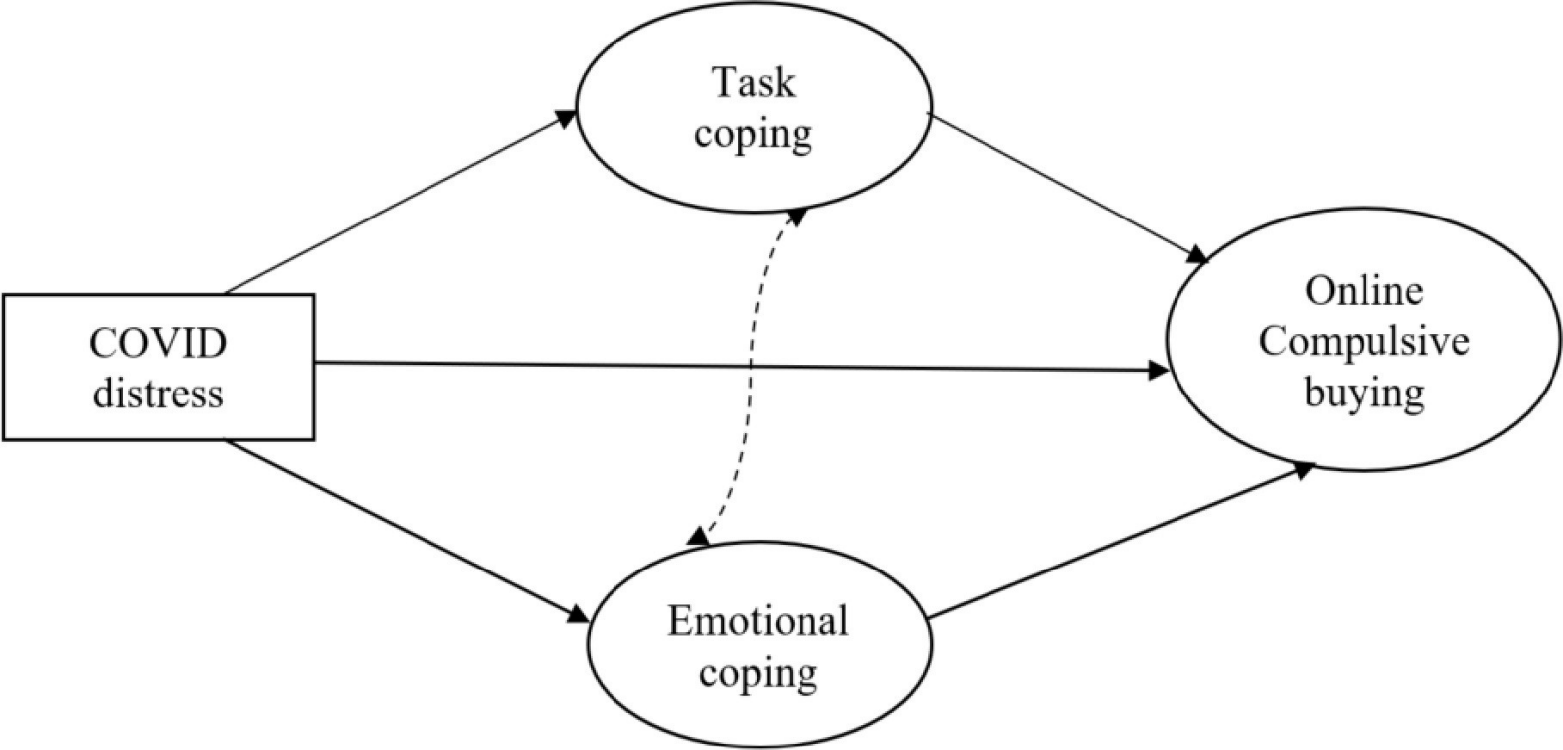
The proposed mediation model.

Our aim was to examine the associations between stress, coping, and compulsive buying during the COVID-19 pandemic. More specifically, in the current study we hypothesized that COVID-related distress would be positively associated with (online) compulsive buying, and that emotion-focused coping, but not task-focused coping, would mediate this association. Avoidance coping was not analyzed in the study, since compulsive buying can be considered as a means of avoidance in itself, while many other activities that may serve as means of avoidance (e.g., meeting friends/family or going out for leisure activities) were restricted by the social distancing orders in place at the time, thus asking whether people were engaging in such activities did not seem rational. The hypothesized associations are presented in Fig 1.

We also explored whether these associations changed as the pandemic evolved. Our previous analysis of this dataset revealed that COVID-related distress among participants oscillated initially, until around day 80 of the pandemic (i.e., around June 1, 2020), when the number of COVID-19 cases started to rise continuously, as did the participants’ COVID-related distress. This steady growth halted at around day 140 of the pandemic (i.e., around July 17), when the number of new confirmed cases peaked in the U.S. Although reported distress began to decline after this peak, the rate of decrease in COVID-related distress was slower than the rate of decrease in the number of COVID cases [30]. At the same time, compulsive buying tendencies were continuously on the rise during our sampling period. Based on these events, we split our data collection into three periods: T1 = day 14-day 80 of the pandemic (03/26/2020–06/01/2020); T2 = day 81–140 of the pandemic (06/02/2020–07/17/2020); T3 = day 141–206 of the pandemic (07/18/2020-10/02/2020). Our goal was to examine whether the mediation models were invariant among these three time periods—that is, whether the associations between COVID-related distress, coping, and compulsive buying were similar over time. To our knowledge, this is the first study to examine whether coping acts as a mediator between distress and compulsive buying during COVID-19.

## Methods

### Sample and procedure

**Fig 2:**
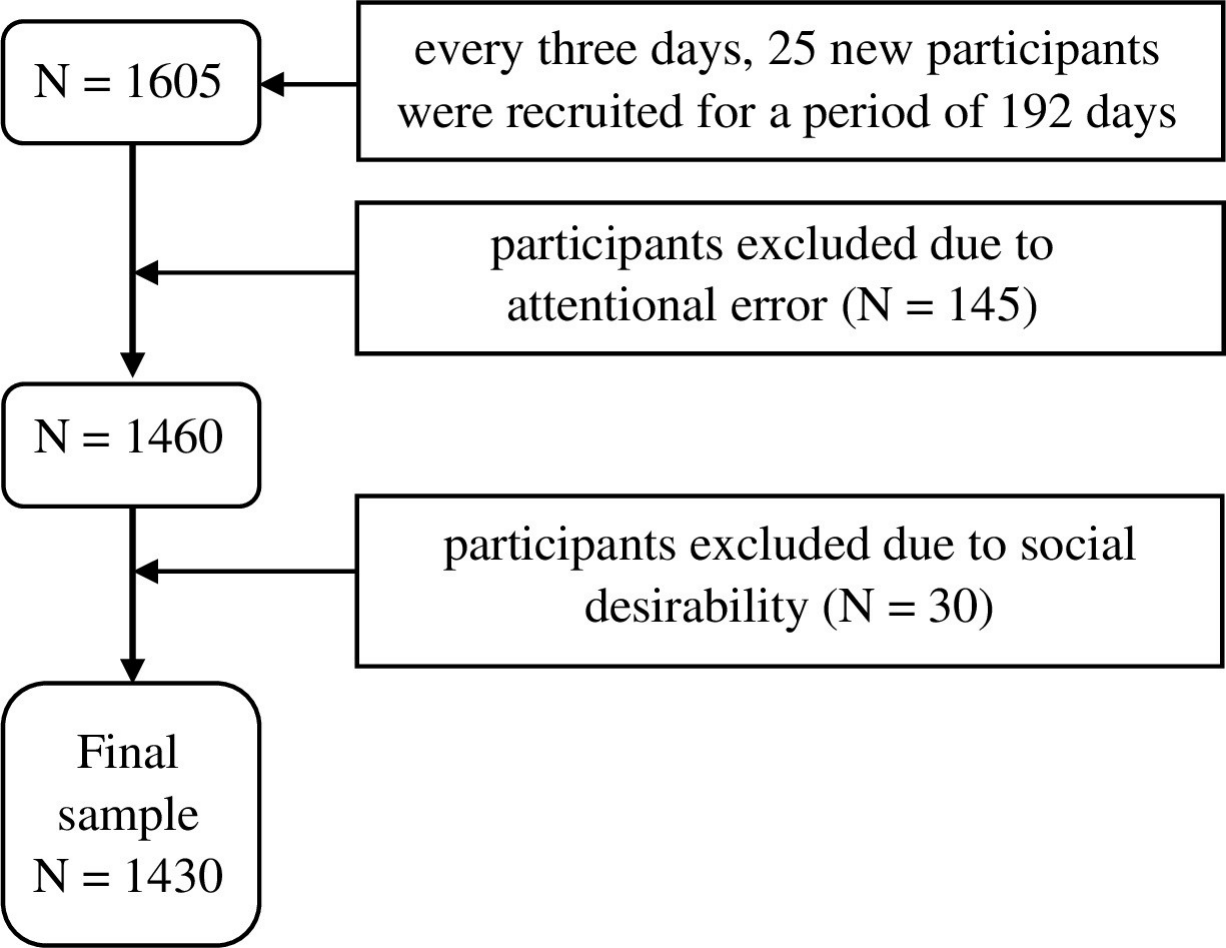
Study participant recruitment process.

Parts of this dataset have been published in two recent studies [30, 35]. Data were collected via Amazon MTurk during the first six months of the COVID-19 pandemic, between March 26, 2020, and October 2, 2020, starting 14 days after the official declaration of the start of the pandemic in the U.S. (Proclamation on Declaring a National Emergency Concerning the Novel Coronavirus Disease [COVID-19] Outbreak, issued on March 13, 2020). Every three days, a new cohort of 25 participants were asked to fill out self-report surveys, excluding individuals who had participated earlier. Inclusion criteria were being 18 years of age or older and logging in from a U.S.-based IP address. Four attention check items were hidden among the regular survey items (e.g., “Please check ‘true’ here.”). Subjects with more than one attentional error were excluded. Furthermore, participants with a maximum score (10 out of 10) on the Marlowe–Crowne Social Desirability Scale [36] were also excluded. The recruitment process is presented in Fig 2.

The final sample of 1,430 was 40% female (N = 572) with a mean age of 36.6 years (SD = 11). The study procedures were in accordance with the Declaration of Helsinki. The informed consent of participants was obtained before the surveys were filled in. The ethical approval of the Institutional Review Board was obtained prior to data collection (2020-15R3).

The study was pre-registered prior to data collection (https://osf.io/m5kw9). The data, materials, and scripts of analysis are available in open access repositories: https://osf.io/qdhp4/ and https://github.com/anikomaraz/shopping_covid19.

### Measures

Subjective socioeconomic status (SES) was assessed with the help of the item *How wealthy do you think you are compared to others*? Participants were asked to respond on a 7-point Likert scale, where 1 corresponded to *Among the poorest*, and 7 corresponded to *Among the richest*.

COVID-related distress was assessed with one item: *How stressed do you feel about the current situation caused by the coronavirus outbreak*? Participants were asked to respond on a 10-point Likert scale ranging from 1 (*Not at all stressed*) to 10 (*Very stressed*).

The short form of the Coping Inventory for Stressful Situations (CISS) [37] is used to assess coping strategies in response to a difficult or stressful situation. Participants were asked to indicate on a 5-point Likert-type scale how they normally react when they encounter a stressful situation, ranging from *Not at all* to *Very much*. Shortened from the original CISS [38], the short form of the CISS is a 21-item self-report survey that consists of three subscales, each comprising seven items: task-focused coping, emotion-focused coping, and avoidance coping via social support or giving oneself treats. Emotion-focused coping, as measured by the CISS, is considered maladaptive due to items related to self-blame or counterfactual thinking, which do not facilitate problem solving and may generate passivity and avoidance [17]. The avoidance coping subscale was not used in the current study due to the fact that most of the activities described in the items in this subscale were restricted during the pandemic (e.g., *Go out for a snack or meal*; *Spend time with a special person*), and due to the obvious overlapping of the item *Buy myself something* with compulsive buying as the outcome measure. Cronbach’s α for task-focused coping and emotion-focused coping in the current sample was 0.828 and 0.883 respectively.

Participants who reported making an offline purchase in the previous seven days were asked to fill out the BERGEN Shopping Addiction Scale (BSAS) [37], which measures offline compulsive buying. The BSAS consists of 28 items that target the Diagnostic and Statistical Manual of Mental Disorders, 5^th^ Edition (DSM-5) [39] addiction criteria of salience, mood modification, conflict, tolerance, relapse, withdrawal, and problems. Participants were asked to respond using a 7-point Likert-type scale (1 = *Strongly disagree*, 7 = *Strongly agree*) to items assessing their shopping behavior in the previous 30 days. Cronbach’s α for the current sample was 0.986.

Online compulsive buying was measured via the Compulsive Online Shopping Scale (COSS) [40]. The COSS was adapted from the BSAS [41] by adding the word “online” to items (e.g., *I felt bad if for some reason I was prevented from shopping/buying things* online). The subscales, the instruction regarding the previous 30 days, and the alternative responses thus corresponded to those described above in case of the BSAS. The minimum and maximum values are identical to the BSAS, ranging from 28 to 140. The COSS was only administered to participants who reported making any purchase online in the previous seven days. Cronbach’s α for the current sample was 0.984.

### Statistical analysis

**Fig 3:**
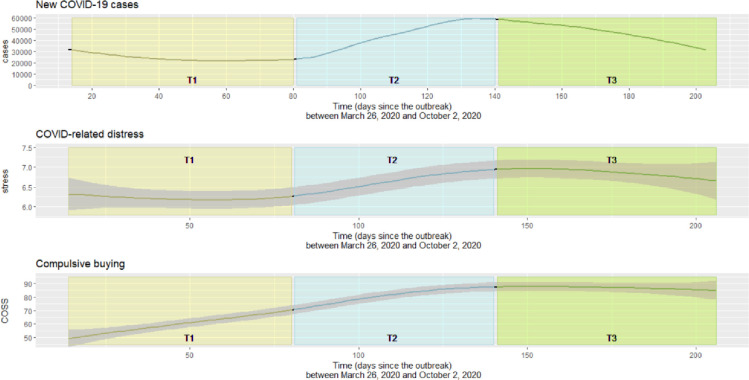
Changes in the number of new COVID cases in the U.S., the level of COVID-related distress, and compulsive buying throughout the period of data collection. Note. COSS = Compulsive Online Shopping Scale. T1 = day 14 –day 80 of the pandemic (03/26/2020–06/01/2020); T2 = day 81 –day 140 of the pandemic (06/02/2020–07/17/2020); T3 = day 141 –day 206 of the pandemic (07/18/2020–10/02/2020). Lines were smoothed to reduce noise in the presentation of the data. Source for the number of new COVID cases in the U.S.: https://www.ecdc.europa.eu/en/publications-data/download-todays-data-geographic-distribution-covid-19-cases-worldwide.

Descriptive statistics and reliability testing were carried out using IBM SPSS 24 software (2016). Structural equation modeling was performed in MPlus software Version 8 [42] with multiple linear regression estimation to test whether the relationship between COVID-related distress and compulsive buying was mediated by emotion-focused and task-focused coping. Gender, age, and subjective SES were controlled for in the model, as previous results for this sample revealed that compulsive buying (both online and offline) differed among high, average, and low SES groups [43] and, according to a meta-analysis, female gender and younger age may be associated with stronger compulsive buying behavior [26]. Coping measures were entered as latent variables (i.e., defined by their individual items), and the outcome measures were entered as parceled variables (i.e., defined by the total scores of the seven subscales). The model was initially estimated for the whole sample (Model 1), and time was subsequently used as a grouping variable (Models 2 & 3). The data were split into three phases, based on major events related to the pandemic in the U.S. that were also reflected in the level of distress and compulsive buying behavior within this sample [30]. These trends are illustrated in Fig 3.

Our goal was to examine whether the mediation models were invariant between the three time periods—that is, whether the associations between COVID-related distress, coping, and compulsive buying were similar over time. We therefore carried out two multigroup mediation analyses, where time was used as a grouping variable. In Model 2, path coefficients were estimated freely, whereas in Model 3 they were constrained to be equal among the three time periods. To test whether the mediations were invariant across time, Model 2 and Model 3 were compared in terms of their chi-square statistics [44]. The proportion mediated—that is, the unstandardized indirect effect divided by the unstandardized total effect [45]—was calculated for each mediation path in T1, T2, and T3. The models were estimated with both online and offline compulsive buying as their outcome measures.

## Results

The means and standard deviations of the assessed variables, as well as their correlations, are presented in Table 1 for the total sample and for T1, T2, and T3 separately.

**Table 1:** Descriptive statistics and Pearson correlations of the assessed measures.

	Total sample (N = 1,430)							T1 (N = 543)							T2 (N = 429)							T3 (N = 455)						
Measure	Mean (SD)	2.	3.	4.	5.	6.	7.	Mean (SD)	2.	3.	4.	5	6	7.	Mean (SD)	2.	3.	4.	5.	6.	7.	Mean (SD)	2.	3.	4.	5.	6.	7.
**1. CISS task**	26.28 (4.97)	-.15**	-.01	-.08**	-.07*	.07**	.10**	26.72 (5.54)	-.27**	-.05	-.15**	-.13*	.095*	.17**	26.34 (4.33)	-.12*	-.05	-.04	-.08	.13**	-.003	25.69 (4.75)	.08	.12*	.06	.06	.04	.06
**2. CISS emot.**	21.28 (6.81)		.46**	.57**	.59**	.18**	-.13**	19.28 (7.32)		.46**	.50**	.48**	.086*	-.21**	22.45 (6.45)		.45**	.63**	.65**	.20**	-.03	22.55 (5.90)		.44**	.49**	.57**	.16**	-.04
**3. COVID distress**	6.56 (2.42)			.40**	.42**	.12**	-.03	6.16 (2.58)			.35**	.34**	-.011	-.01	6.75 (2.36)			.43**	.48**	.22**	-.03	6.84 (2.21)			.38**	.43**	.10*	-.03
**4. COSS**	75.38 (32.81)				.96**	.50**	-.17**	59.12 (27.96)				.94**	.35**	-.22**	83.71 (32.99)				.97**	.56**	-.09	86.01 (30.49)				.96**	.45**	-.11*
**5. BSAS**	74.24 (33.92)					.48**	-.12**	57.92 (29.75)					.37**	-.17**	83.32 (33.45)					.52**	-.03	83.70 (31.94)					.45**	-.06
**6. SES**	4.24 (1.36)						-.08**	3.86 (1.24)						-.10*	4.53 (1.35)						-.06	4.43 (1.40)						-.02
**7. Age**	36.62 (11)							37.82 (11.57)							36.23 (10.73)							35.50 (10.42)						

Note. CISS task = Coping Inventory for Stressful Situations task-focused coping subscale; CISS emot. = Coping Inventory for Stressful Situations emotion-focused coping subscale; COSS = Compulsive Online Shopping Scale; BSAS = Bergen Shopping Addiction Scale; SES = Subjective socioeconomic status.

*Correlations are significant at p < .05.

**Correlations are significant at p < .01.

Notably, there was a significant moderate negative correlation between task-focused and emotion-focused coping in T1, which decreased over T2 and then disappeared in T3. The difference in these correlation coefficients was significant between T1 and T2 (z = -2.41, p < .01), T1 and T3 (z = -5.6, p < .01), as well as T2 and T3 (z = -2.97, p < .01).

There were no significant gender differences in any of the variables. The gender differences in the assessed measures are reported in S1 Table of the Supporting Information.

In our first model, we tested whether the relationship between COVID-related distress and online compulsive buying was mediated by emotion-focused and task-focused coping with the help of structural equation modeling for the entire sample. Age, gender, and subjective SES were controlled for in the mediation model, as there were tendency-level gender differences in emotion-focused coping, and gender and age were significantly correlated to most of the assessed variables. Model 1 demonstrated excellent model fit (χ² = 1117.115, df = 258, RMSEA = 0.048 [0.046–0.051], SRMR = 0.043, CFI = 0.952, TLI = 0.945). As hypothesized, emotion-focused coping mediated the relationship between COVID-related distress (β = 0.47) and compulsive buying (β = 0.47); proportion mediated was 0.32. The explained variance of compulsive buying was 0.56. There was a weak but significant direct path between COVID-related distress and compulsive buying (β = 0.11). However, task-focused coping was not significantly associated with either online compulsive buying or COVID-related distress, thus it was excluded from further analyses.

In Model 2, time was used as a grouping variable to compare the relationship between the assessed variables throughout the different phases of the pandemic. The first period (T1; N = 543) ran from day 14 (the beginning of data collection) to day 80 of the pandemic; the second period (T2; N = 429) started on day 81 and ended on day 140; and the third period (T3; N = 455) ran from day 141 to day 206 (the end of data collection). To assess invariance across these timeframes, two models were estimated. In Model 2, betas were estimated freely, whereas in Model 3, all the path coefficients were constrained to be equal among T1, T2, and T3. Both Model 2 (χ² = 1119.377 [χ²_T1_ = 482.380, χ²_T2_ = 297.892_,_ χ²_T3_ = 339.105]_,_ df = 420, RMSEA = 0.059 [0.055–0.064], SRMR = 0.049, CFI = 0.951, TLI = 0.947) and Model 3 (χ² = 1,146.918 [χ²_T1_ = 496.037, χ²_T2_ = 306.679, χ²_T3_ = 344.201], df = 426, RMSEA = 0.060 [0.056–0.064], SRMR = 0.058, CFI = 0.949, TLI = 0.947) demonstrated excellent model fit. The Satorra–Bentler Scaled Chi-Square Difference Test [44] significantly decreased between Model 2 and Model 3 (TRd = 26.02, Δdf = 6, p < .01), revealing that the models were not invariant across the three time periods. In order to explore when exactly the changes occurred, we conducted post hoc analyses in which path coefficients were constrained one by one, and we compared these to Model 2 (where all betas were estimated freely) using the Satorra–Bentler Scaled Chi-Square Difference Test. These analyses revealed that the path between emotion-focused coping and compulsive buying changed substantially throughout T1, T2, and T3 (TRd = 15.42, Δdf = 2, p < .01). It was moderate during T1 (β = .39, p < .001), increased during T2 (β = .55, p < .001), then decreased during T3 (β = .44, p < .001). The other paths were invariant (TRd = 1.05, Δdf = 2, p = .59 for COVID-related distress and emotion-focused coping; TRd = 3.42, Δdf = 2, p = .18 for COVID-related distress and compulsive buying).

**Fig 4:**
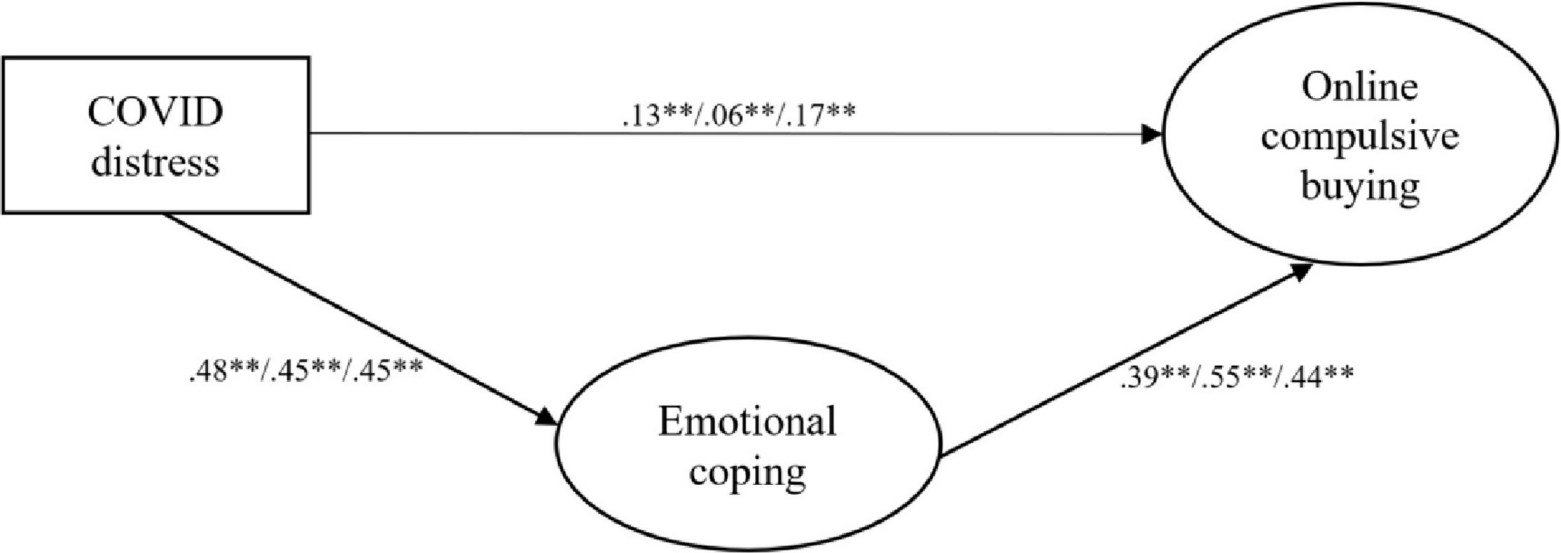
The multi-group mediation model with online compulsive buying as the outcome and its standardized path coefficients for T1, T2, and T3, respectively (Model 2). Note. *p < .01. **p < .001. (χ² = 1119.377 [χ²_T1_ = 482.380, χ²_T2_ = 297.892_,_ χ²_T3_ = 339.105]_,_ df = 420, RMSEA = 0.059 [0.055–0.064], SRMR = 0.049, CFI = 0.951, TLI = 0.947).

As shown in Fig 4, there was a weak but significant path between COVID-related distress and compulsive buying. The relationship between COVID-related distress and compulsive buying was mediated by emotion-focused coping across the three time periods—that is, higher COVID-related distress was associated with a more frequent use of emotion-focused coping, which in turn was associated with more compulsive buying online. The standardized indirect effect was 0.19 (p < .001), 0.25 (p < .001), and 0.20 (p < .001) for T1, T2, and T3 respectively. Proportion mediated for COVID-related distress, emotion-focused coping, and online compulsive buying was 59.45% across the three time periods. Subjective SES, age, and gender were controlled for in the model, where higher subjective SES was significantly associated with more compulsive buying (β_T1_ = .355, p < .001, β _T2_ = .424, p < .001, β _T3_ = .387 p < .001). The total explained variance of online compulsive buying was 39.7% (p < .001) in T1, 64.1% (p < .001) in T2, and 51.6% (p < .001) in T3.

We repeated these analyses with offline compulsive buying as the outcome measure, obtaining almost identical results (time invariance). The multigroup mediation model for offline compulsive buying and its fit indices are available in S1 Fig in the Supporting Information.

## Discussion

Our findings suggest that the relationship between stress and compulsive buying is mediated by maladaptive coping during the COVID-19 pandemic. Furthermore, our results indicate that interindividual differences in the tendency to employ maladaptive emotion-focused coping strategies such as self-blame, counterfactual thinking, or becoming very upset in response to COVID-related distress may further exacerbate stress and give rise to maladaptive behavioral outcomes such as compulsive buying. Compulsive buying may be a source of stress itself, which may require further coping efforts on top of the already stressful pandemic, resulting in a vicious circle [46]. This highlights that the response to a stressor may play a significant role in adaptation [7, 8] and underscores the importance of psychoeducation/counselling on adaptive ways of coping, especially during challenging times such as the COVID-19 pandemic.

The mediation model was not invariant across the three time periods, indicating that the strength of the associations between COVID-related distress, emotion-focused coping, and compulsive buying was not the same across T1, T2, and T3. Specifically, the relationship between emotion-focused coping as the mediator and compulsive buying as the outcome was moderate during T1, became stronger during T2, and then decreased again during T3. One reason for this may be that buying tendencies have varied throughout the pandemic—for instance, people initially hoarded certain products either in an attempt to avoid catching the virus (e.g., hand sanitizers, face masks) or due to panic about potential supply shortages (e.g., food, toilet paper) [47]. However, the connection between COVID-related distress, emotion-focused coping, and compulsive buying persisted beyond the first period of panic. Moreover, the associations between emotion-focused coping and compulsive buying grew stronger in T2, when these initial buying behaviors became less salient. This suggests that there is an emotional aspect to compulsive buying, rather than it being hoarding behavior or simply overbuying. In fact, stress and buying tendencies fluctuated, but were generally on the rise during the time of data collection [30], while emotion-focused coping increased from T1 to T2 and stagnated for the rest of our sampling period. The increased tendency to employ an emotion-focused, often self-blaming coping strategy may reflect the fact that by this time (day 80 –day 141, June 2 to July 17, 2020) people had become exhausted by the demands and the unfamiliar circumstances generated by the pandemic. Compulsive buying played the strongest role in terms of coping with distress during the period when distress was on the rise (T2), as indicated by the largest proportion of variance explained within this period and the fact that the path between emotional coping and compulsive buying was strongest during this time.

Another interesting finding that suggests a potential change in coping profiles is that the correlations between task-focused and emotion-focused coping changed substantially across the three time periods. Initially, there was a moderate negative association, which decreased over time and then disappeared. This may suggest that, immediately after the outbreak of the pandemic, people tended to commit to one or another coping strategy, whereas these boundaries became less articulated as time passed. Task-focused coping was not associated with COVID-related distress, which may reflect the notion that perceived stress regarding the pandemic fosters emotion-focused rather than action-oriented, task-focused coping strategies, or that task-focused coping strategies may be more effective in reducing COVID-related stress. However, we need to be cautious when interpreting the changes that occurred over time. Given that our study has a cross-sectional design, we cannot draw conclusions about intraindividual changes in coping styles. Specifically, based on our results it is not possible to determine whether the observed differences between T1, T2, and T3 lie in the differences in the recruited samples or truly reflect different phases in people’s reactions to the unfolding pandemic. Our results raise the question of whether the pandemic, as a persistent stressor, fosters changes in coping profiles over time, but this question can only be answered by a longitudinal study.

In the same vein, one might argue for a reversed model, assuming that maladaptive emotion-focused coping may be associated with elevated stress, which, in turn, may lead to more compulsive buying. We tested this alternative multigroup model, in which emotion-focused coping was the predictor and COVID-related distress was the mediator, for both online and offline compulsive buying. Although these alternative models also demonstrated excellent model fit, we argue that our proposed model makes more sense conceptually. Emotion-focused coping was assessed as a trait-like person-level variable, whereas stress was assessed as a state-level variable, as we were interested in the time-varying effects of stress throughout the initial phase of the pandemic. We therefore posit that it is more plausible for a state-like variable (COVID-related distress) to trigger a trait-like variable (emotion-focused coping), which in turn affects the behavioral outcome, than vice versa.

The strengths of this research include the fast reaction to the pandemic (data collection started two weeks after the outbreak of the pandemic in the U.S.); data collection that was spread over time, which enabled us to compare our mediation model in different phases of the pandemic; and the large sample size. However, certain limitations must be taken into consideration. We collected the self-reported data cross-sectionally only, thus the data are prone to response bias and do not allow us to draw causal conclusions. Furthermore, we assessed general coping styles in a stressful situation rather than the actual coping strategies employed in response to the pandemic. Although it is reasonable to assume that general coping styles are likely to be adopted across different stressful situations, the fact that we did not assess other coping strategies used in response to COVID-related distress is a limitation of our study. Another limitation is that compulsive buying itself is sometimes considered as a means of avoidance coping [48], making it hard to distinguish our outcome variable from our mediators.

In summary, our results indicate that maladaptive emotion-focused coping, including self-blame and counterfactual thinking, mediated the relationship between COVID-related distress and compulsive buying behavior. In other words, interindividual differences in coping may play a substantial role in the occurrence of maladaptive behavioral outcomes such as compulsive buying. At the time of the COVID-19 pandemic, when various coping strategies (e.g., social support, going out to engage in leisure activities) are hampered, people may seek other activities to disengage from COVID-related distress. Compulsive buying may represent one such attempt, especially when maladaptive emotion-focused coping is used. It is therefore important to promote active coping strategies that are more adaptive in terms of stress reduction via psychoeducation, especially during challenging times such as the COVID-19 pandemic. Such strategies might include finding new hobbies or interests that can be practiced indoors, or performing socially distanced outdoor activities (e.g., going biking or taking walks in the neighborhood) as a means of distraction from COVID-related distress.

## Supporting information

S1 Table Gender differences of the assessed measures. Note. CISS task = Coping Inventory for Stressful Situations task-focused coping subscale; CISS emot. = Coping Inventory for Stressful Situations emotion-focused coping subscale; COSS = Compulsive Online Shopping Scale; BSAS = Bergen Shopping Addiction Scale; SES = Subjective socio-economic status.
(DOCX)

S1 Fig The multi-group mediation model with offline compulsive buying as the outcome and its standardized path coefficients for T1, T2 and T3, respectively. Note. *p < .01. **p < .001. χ² = 955.107 [χ²_T1_ = 377.307, χ²_T2_ = 255.708, χ²_T3_ = 322.092], df = 420, RMSEA = 0.052 [0.048–0.056], SRMR = 0.044, CFI = 0.961, TLI = 0.960. Standardized indirect effect was 0.188 (p < 0.001), 0.253 (p < 0.001) and 0.218 (p < 0.001) for T1, T2 and T3 respectively. Proportion mediated for COVID-related distress, emotion-focused coping and offline compulsive buying was 57.5% across the three time periods. The total explained variance of online compulsive buying was 39.1% (p < .001) in T1, 66.3% (p < .001) in T2, and 53.2% (p < .001) in T3.
(DOCX)
