# Supplementary material for: Emotion-focused coping mediates the relationship between COVID-related distress and compulsive buying
Source: PLoS One. 2022 Sep 15;17(9):e0274458. doi: 10.1371/journal.pone.0274458 (PMC9477291; doi:10.1371/journal.pone.0274458)
Supplement: S1 Table — Note. CISS task = Coping Inventory for Stressful Situations task-focused coping subscale; CISS emot. = Coping Inventory for Stressful Situations emotion-focused coping subscale; COSS = Compulsive Online Shopping Scale; BSAS = Bergen Shopping Addiction Scale; SES = Subjective socio-economic status. (DOCX) [file pone.0274458.s001.docx]

**SUPPORTING INFORMATION**

S1 Table. Gender differences of the assessed measures.

| **Measure** | **Mean Male (SD)**  N= 858 | **Mean Female (SD)**  N= 572 | **t-value** | **p** | **Cohen’s d** |
| --- | --- | --- | --- | --- | --- |
| **1. CISS task** | 26.22 (5.00) | 26.37 (4.91) | .548 | .58 | 0.003 |
| **2. CISS emot.** | 21.61 (6.86) | 21.01 (6.75) | 1.645 | .10 | 0.009 |
| **3. COVID distress** | 6.63 (2.37) | 6.51 (2.46) | .872 | .38 | 0.049 |
| **4. COSS** | 74.09 (31.94) | 76.49 (33.42) | 1.203 | .23 | 0.007 |
| **5. BSAS** | 72.56 (33.30) | 75.64 (34.31) | 1.467 | .14 | 0.009 |
| **6. SES** | 4.24 (1.35) | 4.26 (1.35) | .274 | .78 | 0.001 |

Note. CISS task = Coping Inventory for Stressful Situations task-focused coping subscale; CISS emot. = Coping Inventory for Stressful Situations emotion-focused coping subscale; COSS = Compulsive Online Shopping Scale; BSAS = Bergen Shopping Addiction Scale; SES = Subjective socio-economic status.
