# Supplementary material for: Emotion-focused coping mediates the relationship between COVID-related distress and compulsive buying
Source: PLoS One. 2022 Sep 15;17(9):e0274458. doi: 10.1371/journal.pone.0274458 (PMC9477291; doi:10.1371/journal.pone.0274458)
Supplement: S1 Fig — Note. *p < .01. **p < .001. χ² = 955.107 [χ²T1 = 377.307, χ²T2 = 255.708, χ²T3 = 322.092], df = 420, RMSEA = 0.052 [0.048–0.056], SRMR = 0.044, CFI = 0.961, TLI = 0.960. Standardized indirect effect was 0.188 (p < 0.001), 0.253 (p < 0.001) and 0.218 (p < 0.001) for T1, T2 and T3 respectively. Proportion mediated for COVID-related distress, emotion-focused coping and offline compulsive buying was 57.5% across the three time periods. The total explained variance of online compulsive buying was 39.1% (p < .001) in T1, 66.3% (p < .001) in T2, and 53.2% (p < .001) in T3. (DOCX) [file pone.0274458.s002.docx]

**SUPPORTING INFORMATION**

S1 Figure. The multi-group mediation model with offline compulsive buying as the outcome and its standardized path coefficients for T1, T2 and T3, respectively.

COVID distress

.14**/.12**/.16**

.48**/.45**/.45**

.39**/.56**/.48**

Note. *p < .01. **p < .001. χ²= 955.107 [χ²_T1_= 377.307, χ²_T2_= 255.708, χ²_T3_= 322.092], df = 420, RMSEA = 0.052 [0.048-0.056], SRMR = 0.044, CFI = 0.961, TLI = 0.960. Standardized indirect effect was 0.188 (p < 0.001), 0.253 (p < 0.001) and 0.218 (p < 0.001) for T1, T2 and T3 respectively. Proportion mediated for COVID-related distress, emotion-focused coping and offline compulsive buying was 57.5% across the three time periods. The total explained variance of online compulsive buying was 39.1% (p < .001) in T1, 66.3% (p < .001) in T2, and 53.2% (p < .001) in T3.
